# LINC01410 accelerated the invasion and proliferation of osteosarcoma by sponging miR-3128

**DOI:** 10.18632/aging.103464

**Published:** 2020-12-19

**Authors:** Quanxiao Xu, Limin He, Lei Ma, Lin Fan, Lihua Yan, Xulin Zhao, Yuanyuan Li

**Affiliations:** 1 Henan Medical Key Laboratory of Tumor Molecular Biology, Nanyang First People’s Hospital, Nanyang 473012, Henan Province, China; 2 Department of Medical Oncology, Nanyang Second People's Hospital, Nanyang 473000, Henan, China

**Keywords:** LINC01410, miR-3128, osteosarcoma, oncogene

## Abstract

Increasing evidence has shown that lncRNAs are closely correlated with cell apoptosis, autophagy and progression. However, the role of LINC01410 in osteosarcoma has not been verified. We determined that LINC01410 was overexpressed in osteosarcoma specimens and cell lines. The expression of LINC01410 was upregulated in 22 osteosarcoma patients (22/30, 73%) compared to control normal samples. Ectopic expression of LINC01410 promoted the osteosarcoma cell cycle, proliferation and invasion. Overexpression of LINC01410 induced N-cadherin and Vimentin expression and inhibited E-cadherin expression in osteosarcoma cells. LINC01410 acted as a sponge for miR-3128. The results showed that miR-3128 overexpression decreased the luciferase activity of WT-LINC01410 but not mut-LINC01410 in MG-63 cells. Upregulation of LINC01410 expression suppressed miR-3128 expression in MG-63 cells. Moreover, LINC01410 overexpression increased osteosarcoma cell invasion and growth by modulating miR-3128. These data indicated that LINC01410 acted as an oncogene in osteosarcomagenesis and might be a potential new strategy for osteosarcoma treatment.

## INTRODUCTION

Osteosarcoma is a primary mesenchymal neoplasm and is the major cause of tumor-related death in adolescents and children [1–5]. Osteosarcoma usually occurs in long bones, including the femur, humerus and tibia, in 80% of patients [6–10]. With improvements in osteosarcoma therapy, the five-year survival rate of osteosarcoma cases with metastatic disease remains unsatisfactory [11–14]. However, the fundamental mechanism of metastasis development and drug resistance in osteosarcoma is poorly understood [15–17]. Thus, it is essential to better understand the mechanism of osteosarcomagenesis development and progression to find new strategies for the prognosis, treatment and diagnosis of cases with osteosarcoma.

LncRNAs are a category of nonprotein coding RNAs longer than two hundred nucleotides [18–21]. LncRNAs are increasingly considered important molecules in modulating diverse biological processes, such as metabolism, apoptosis, differentiation and invasion [22–25]. LncRNAs have been indicated to be deregulated in different tumors, including nasopharyngeal carcinoma, gallbladder carcinoma, melanoma, gastric carcinoma and osteosarcoma [26–29]. Recently, LINC01410 was identified as an oncogene in cholangiocarcinoma, colon tumor, gastric cancer and thyroid carcinoma [30–33]. However, its functional role in the progression and tumorigenesis of osteosarcoma remains unclear.

We determined that LINC01410 was overexpressed in osteosarcoma specimens and cell lines and that ectopic expression of LINC01410 induced cell invasion and growth by regulating miR-3128 in osteosarcoma.

## RESULTS

### LINC01410 was overexpressed in osteosarcoma specimens and cells

**Figure 1:**
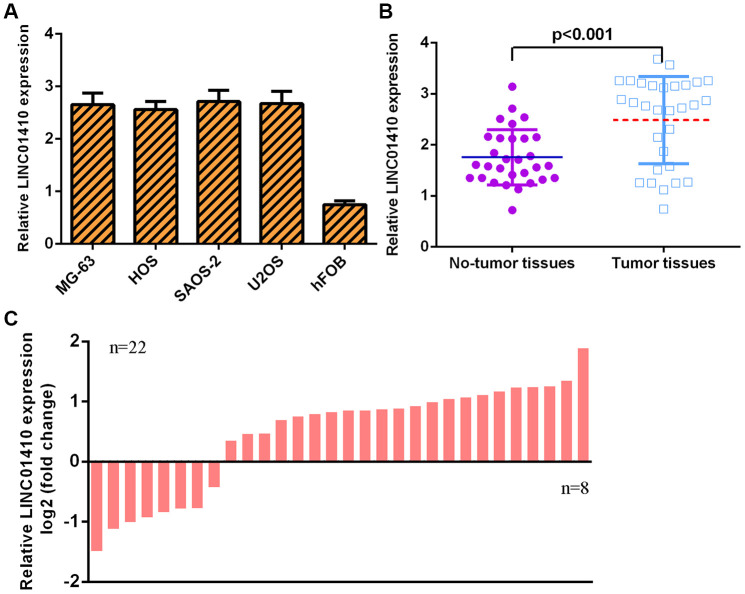
**LINC01410 was overexpressed in osteosarcoma specimens and cells.** (**A**) The expression of LINC01410 in osteosarcoma cells (MG-63, HOS, SAOS-2 and U2OS) and osteoblast cells (hFOB1.19) was detected by qRT-PCR analysis. (**B**) LINC01410 levels were higher in osteosarcoma specimens than in control normal samples. (**C**) The expression of LINC01410 was overexpressed in 22 osteosarcoma patients compared to control normal samples.

LINC01410 expression was first investigated in osteosarcoma cells. We found that LINC01410 was overexpressed in osteosarcoma cells (MG-63, HOS, SAOS-2 and U2OS) compared with osteoblast cells (hFOB1.19) (Figure 1A). The LINC01410 level was higher in osteosarcoma specimens than in control normal samples (Figure 1B). The expression of LINC01410 was upregulated in 22 osteosarcoma patients compared to control normal samples (Figure 1C).

### miR-3128 was decreased in osteosarcoma specimens and cells

**Figure 2:**
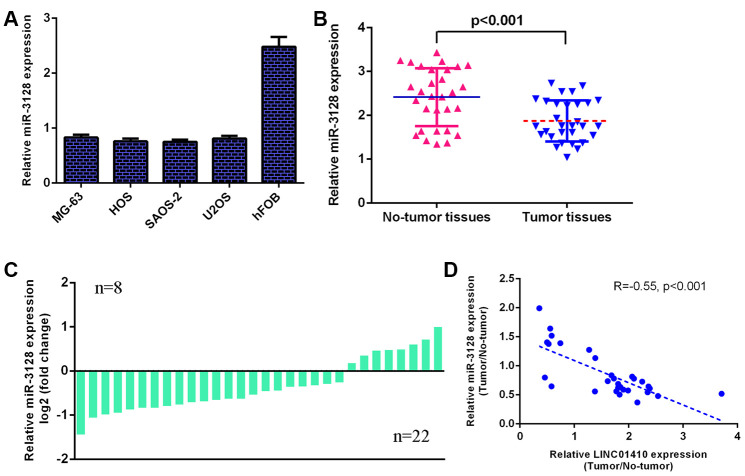
**miR-3128 was decreased in osteosarcoma specimens and cells.** (**A**) The expression of miR-3128 in osteosarcoma cells (MG-63, HOS, SAOS-2 and U2OS) and osteoblast cells (hFOB1.19) was detected by qRT-PCR analysis. (**B**) miR-3128 levels were lower in osteosarcoma specimens than in control normal samples. (**C**) The expression of miR-3128 was downregulated in 22 osteosarcoma patients compared to control normal samples. (**D**) There is an inverse correlation between miR-3128 and LINC01410 in osteosarcoma specimens.

Furthermore, miR-3128 expression was investigated in osteosarcoma cells. We determined that miR-3128 was decreased in osteosarcoma cells (MG-63, HOS, SAOS-2 and U2OS) compared with osteoblast cells (hFOB1.19) (Figure 2A). The miR-3128 level was lower in osteosarcoma specimens than in control normal samples (Figure 2B). The expression of miR-3128 was downregulated in 22 osteosarcoma patients compared to control normal samples (Figure 2C). There was an inverse correlation between miR-3128 and LINC01410 in osteosarcoma specimens (Figure 2D).

### LINC01410 overexpression induced osteosarcoma cell growth and cell cycle progression

**Figure 3:**
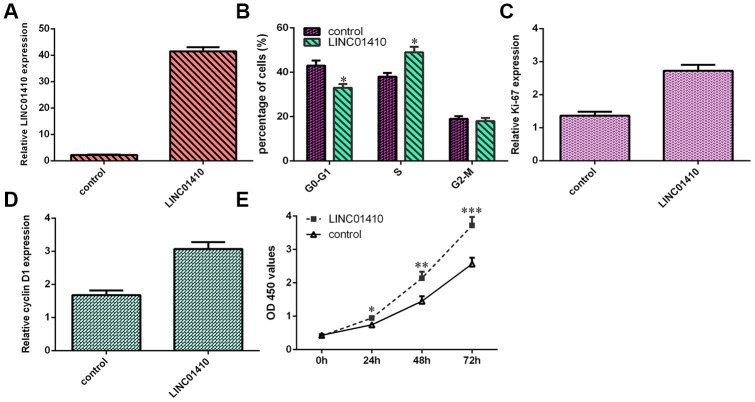
**LINC01410 overexpression induced osteosarcoma cell growth and cell cycle progression.** (**A**) The expression of LINC01410 was detected by qRT-PCR analysis. (**B**) Cell cycle analysis indicated that overexpression of LINC01410 promoted the MG-63 cell cycle. (**C**) Ectopic LINC01410 expression increased ki-67 expression in MG-63 cells. (**D**) Cyclin D1 expression was measured by qRT-PCR. (**E**) Overexpression of LINC01410 enhanced the growth of MG-63 cells. *p<0.05, **p<0.01 and ***p<0.001.

To determine the function of LINC01410, pcDNA-LINC01410 was utilized to enhance LINC01410 expression in MG-63 cells (Figure 3A). Subsequently, cell cycle analysis indicated that overexpression of LINC01410 promoted the MG-63 cell cycle (Figure 3B). Ectopic LINC01410 expression increased ki-67 expression in MG-63 cells (Figure 3C). Elevated expression of LINC01410 promoted cyclin D1 expression in MG-63 cells (Figure 3D). Overexpression of LINC01410 enhanced the growth of MG-63 cells (Figure 3E).

### Ectopic expression of LINC01410 promoted osteosarcoma cell invasion

**Figure 4:**
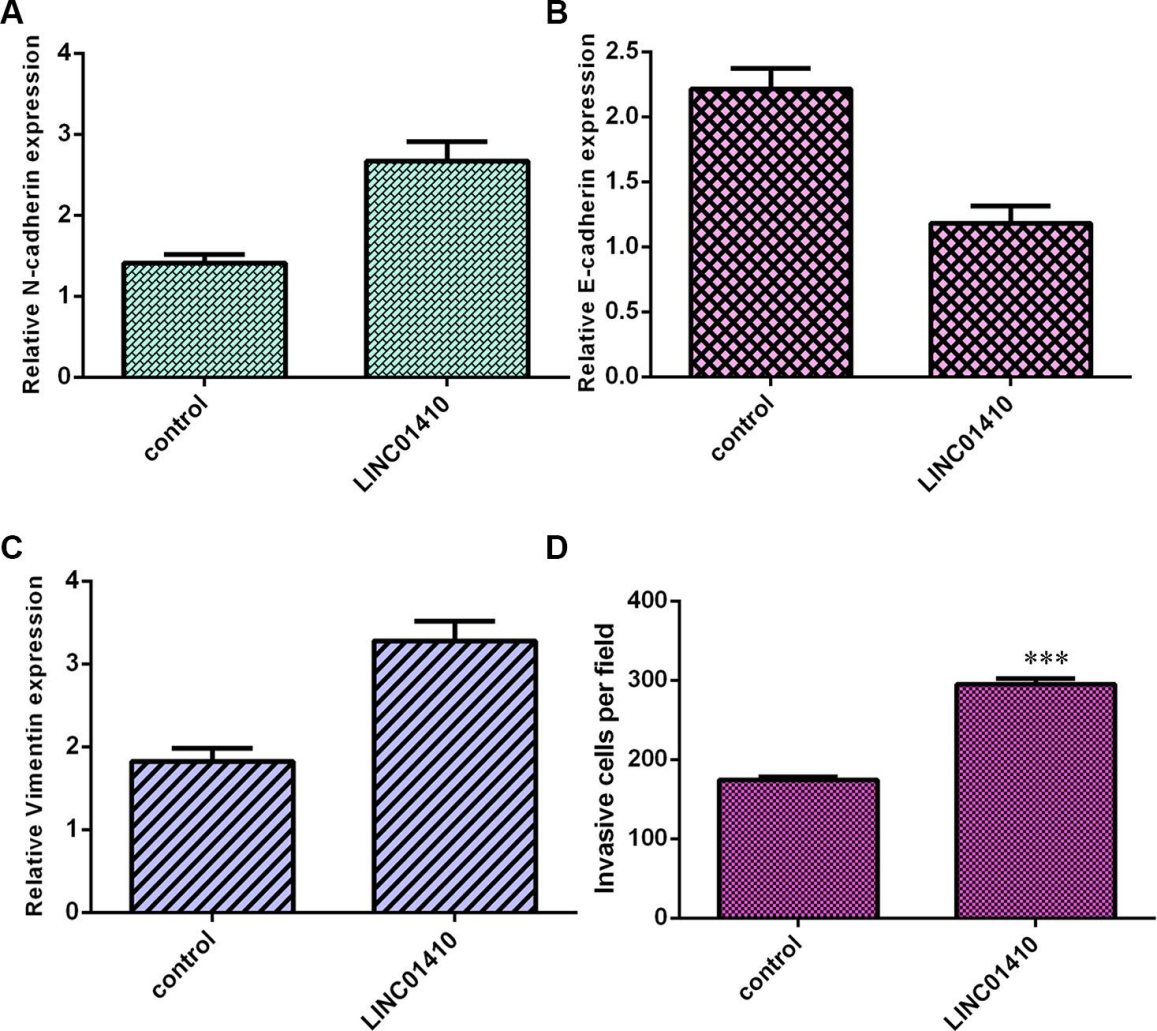
**Ectopic expression of LINC01410 promoted osteosarcoma cell invasion.** (**A**) Elevated expression of LINC01410 induced N-cadherin expression in MG-63 cells. (**B**) The expression of E-cadherin was analyzed by qRT-PCR assay. (**C**) Ectopic expression of LINC01410 promoted Vimentin expression in MG-63 cells. (**D**) Elevated expression of LINC01410 increased invasion in MG-63 cells. ***p<0.001.

Next, elevated expression of LINC01410 was found to induce N-cadherin expression in MG-63 cells (Figure 4A). LINC01410 overexpression promoted E-cadherin expression in MG-63 cells (Figure 4B). In addition, ectopic expression of LINC01410 promoted Vimentin expression in MG-63 cells (Figure 4C). Elevated expression of LINC01410 increased invasion in MG-63 cells, and the relative number of invasive cells is shown (Figure 4D).

### LINC01410 acted as a sponge for miR-3128

**Figure 5:**
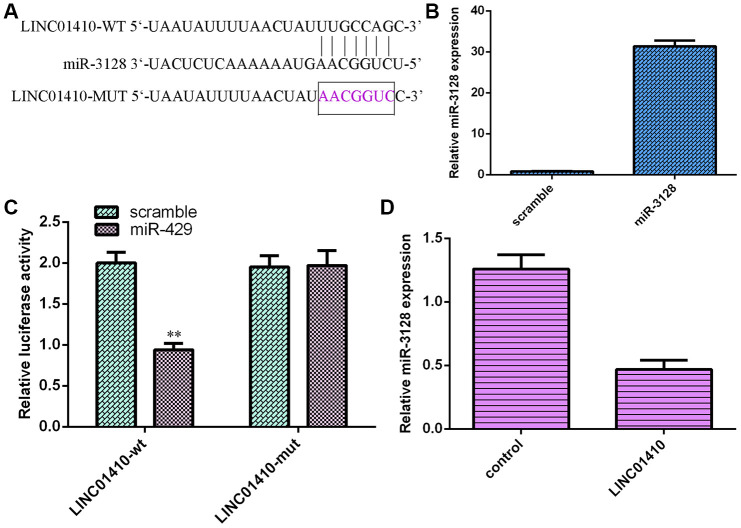
**LINC01410 acted as a sponge for miR-3128.** (**A**) LINC01410 was predicted to have binding sites for miR-3128 by using StarBase. (**B**) miR-3128 expression was detected by qRT-PCR assay. (**C**) miR-3128 overexpression decreased the luciferase activity of WT-LINC01410 but not mut-LINC01410 in MG-63 cells. (**D**) Upregulation of LINC01410 expression suppressed miR-3128 expression in MG-63 cells. **p<0.01.

LINC01410 was predicted to have binding sites for miR-3128 by using StarBase (Figure 5A). miR-3128 expression was upregulated in MG-63 cells after transfection with the miR-3128 mimic (Figure 5B). To investigate the hypothesis, the luciferase reporter assay was performed. The results illustrated that miR-3128 overexpression decreased the luciferase activity of WT-LINC01410 but not mut-LINC01410 in MG-63 cells (Figure 5C). Upregulation of LINC01410 expression suppressed miR-3128 expression in MG-63 cells (Figure 5D).

### LINC01410 overexpression increased osteosarcoma cell invasion and growth by modulating miR-3128

**Figure 6:**
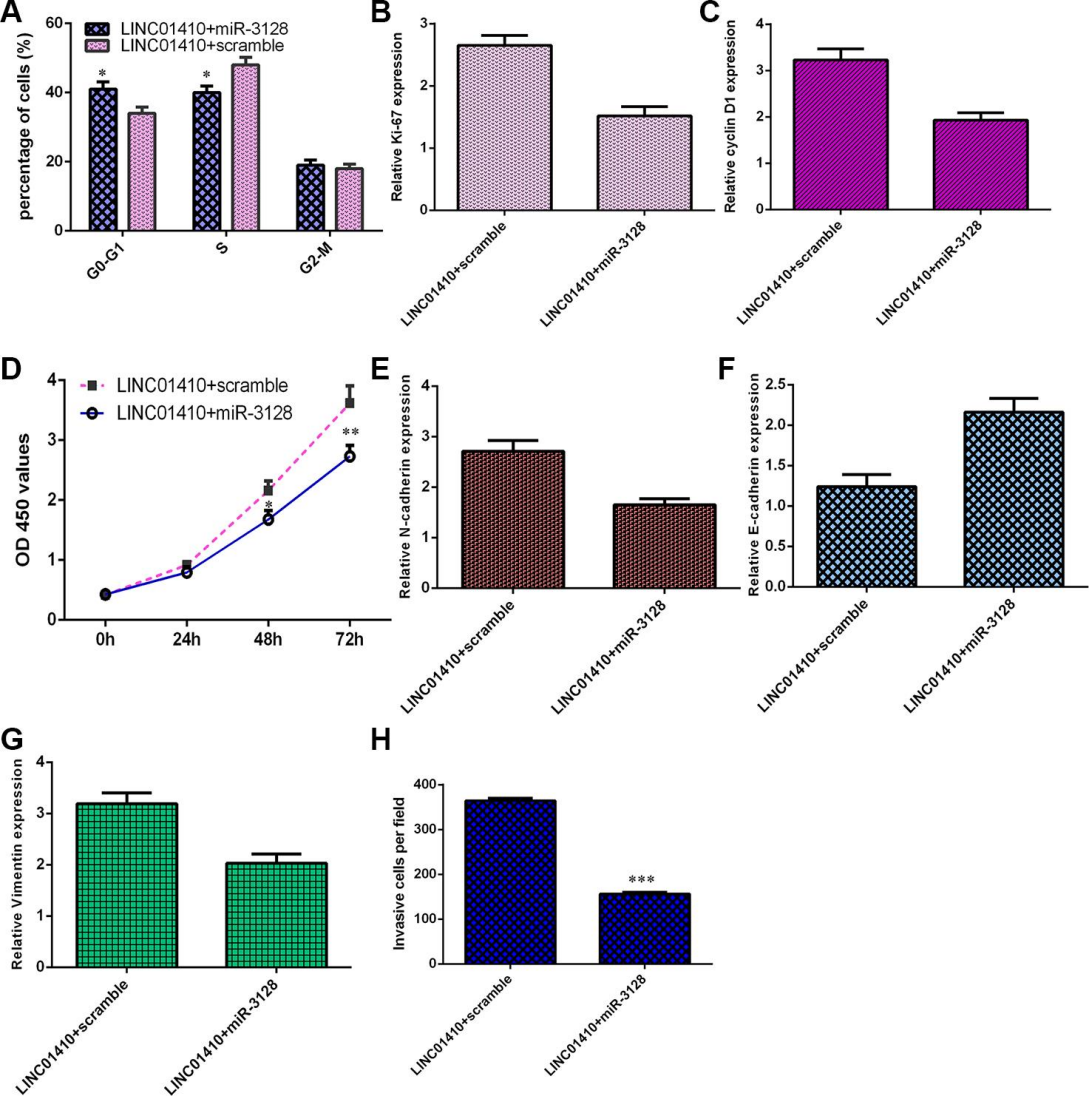
**LINC01410 overexpression increased osteosarcoma cell invasion and growth by modulating miR-3128.** (**A**) Ectopic expression of miR-3128 inhibited the cell cycle in LINC01410-overexpressing MG-63 cells. (**B**) The expression of ki-67 was analyzed by qRT-PCR assay. (**C**) The expression of cyclin D1 was analyzed by qRT-PCR assay. (**D**) Overexpression of miR-3128 decreased the proliferation of LINC01410-overexpressing MG-63 cells. (**E**) The expression of N-cadherin was analyzed by qRT-PCR assay. (**F**) The expression of E-cadherin was analyzed by qRT-PCR assay. (**G**) The expression of Vimentin was analyzed by qRT-PCR assay. (**H**) The relative number of invasive cells is shown. *p<0.05, **p<0.01 and ***p<0.001.

To explore whether LINC01410 plays an oncogenic role in osteosarcoma by regulating miR-3128, we transfected LINC01410-overexpressing MG-63 cells with a miR-3128 mimic. As indicated in Figure 6A, ectopic expression of miR-3128 inhibited the cell cycle in LINC01410-overexpressing MG-63 cells. Elevated expression of miR-3128 suppressed ki-67 (Figure 6B) and cyclin D1 (Figure 6C) in LINC01410-overexpressing MG-63 cells. Overexpression of miR-3128 decreased the proliferation of LINC01410-overexpressing MG-63 cells (Figure 6D). Elevated expression of miR-3128 suppressed N-cadherin (Figure 6E) and Vimentin (Figure 6G) expression and enhanced E-cadherin expression (Figure 6F) in LINC01410-overexpressing MG-63 cells. Finally, miR-3128 overexpression inhibited cell invasion in LINC01410-overexpressing MG-63 cells, and the relative number of invasive cells is shown (Figure 6H).

## DISCUSSION

A growing number of reports have suggested that lncRNA expression is deregulated in many tumors and that lncRNAs play critical roles in cell metabolism, apoptosis, differentiation and invasion. In our study, LINC01410 was overexpressed in osteosarcoma specimens and cell lines. The expression of LINC01410 was upregulated in 22 osteosarcoma patients (22/30, 73%) compared to control normal samples. Ectopic expression of LINC01410 promoted the osteosarcoma cell cycle, proliferation and invasion. Overexpression of LINC01410 induced N-cadherin and Vimentin expression and inhibited E-cadherin expression in osteosarcoma cells. LINC01410 acted as a sponge for miR-3128. This finding illustrated that miR-3128 overexpression decreased the luciferase activity of WT-LINC01410 but not mut-LINC01410 in MG-63 cells (Figure 5C). Upregulation of LINC01410 expression suppressed miR-3128 expression in MG-63 cells. Moreover, LINC01410 overexpression increased osteosarcoma cell invasion and growth by modulating miR-3128. These data indicated that LINC01410 acted as an oncogene in osteosarcomagenesis and might be a potential new strategy for osteosarcoma treatment.

Previous studies revealed that LINC01410 is an oncogene in cholangiocarcinoma, colon tumor, gastric cancer and thyroid carcinoma [30–33]. Jiang et al. [33]. found that LINC01410 was overexpressed in cholangiocarcinoma cells and specimens and that LINC01410 deficiency suppressed cell invasion, migration and growth by targeting miR-124-3p/SMAD5. Wang et al. [32]. showed that LINC01410 was elevated in thyroid tumors and that knockdown of LINC01410 facilitated apoptosis and inhibited growth in thyroid tumors by modulating miR-3619-5p/FOXM1. Luo and colleagues indicated that LINC01410 was highly expressed in colon cancer specimens and that knockdown of LINC01410 reduced cell invasion, growth and cell cycle progression [31]. It has been shown that LINC01410 overexpression induces gastric cancer metastasis and angiogenesis via NF-κB [30]. Increasing evidence has also revealed that lncRNAs play crucial roles in osteosarcomagenesis [34]. Gui et al. [35]. revealed that CDKN2B-AS1 was elevated in osteosarcoma and that CDKN2B-AS1 knockdown inhibited EMT progression, migration and invasion via miR-4458. Hou et al. [36]. indicated that SNHG14 was overexpressed in osteosarcoma specimens and cells and that SNHG14 knockdown suppressed cell growth and promoted cell apoptosis through miR-433-3p. Sun et al. [37]. demonstrated that XIST was elevated in osteosarcoma specimens and cells and that XIST knockdown suppressed osteosarcoma cell autophagy and proliferation and enhanced apoptosis by regulating miR-375-3p. Our study indicated that LINC01410 acted as an oncogene in osteosarcomagenesis.

Previous research proved that lncRNAs modulated multiple cell biological processes by sponging miRNAs. For instance, Wang et al. [38]. indicated that HOXA-AS2 knockdown suppressed cell invasion, viability and migration via E2F3/miR-124-3p in osteosarcoma cells. Li et al. [39]. revealed that NR2F1-AS1 promoted osteosarcoma progression by sponging miR-483-3p. Jin et al. [40]. demonstrated that SND1-IT1 enhanced osteosarcoma migration and growth by regulating miRNA-665. Luo and colleagues indicated that ADPGK-AS1 modulated osteosarcoma cell migration, proliferation, apoptosis and invasion by sponging miR-542-3p [41]. Furthermore, LINC01410 regulated colon cancer cell invasion, growth and cell cycle progression by sponging miR-3128 [31]. We used StarBase to show that LINC01410 was predicted to have binding sites for miR-3128. Luciferase reporter assay data illustrated that miR-3128 overexpression decreased the luciferase activity of WT-LINC01410 but not mut-LINC01410 in MG-63 cells. In addition, we showed that miR-3128 was decreased in osteosarcoma specimens and cells and that there was an inverse correlation between miR-3128 and LINC01410 in osteosarcoma specimens. Furthermore, LINC01410 overexpression increased osteosarcoma cell invasion and growth by modulating miR-3128.

In summary, we determined that LINC01410 was overexpressed in osteosarcoma specimens and cell lines and that ectopic expression of LINC01410 induced cell invasion and growth by regulating miR-3128 in osteosarcoma. These results suggest that LINC01410 acts as an oncogene in osteosarcomagenesis and might be a potential new strategy for osteosarcoma treatment.

## MATERIALS AND METHODS

### Specimens, cell culture and transfection

Thirty pairs of osteosarcoma specimens and control samples were collected at our hospital. Specimens were collected with informed patient consent, and our research was approved by the Clinical Ethical Committee of Nanyang First People’s Hospital. Osteosarcoma cell lines (MG-63, HOS, SAOS-2 and U2OS) and normal osteoblast cells (hFOB1.19) were used in our research. pcDNA-control, pcDNA-LINC01410, scramble and miR-3128 mimic were purchased from GenePharma (Shanghai, China). Cell transfection was carried out using Lipofectamine 2000 (Invitrogen Inc.)

### Real-time PCR assay

Cellular RNA was collected using a TRIzol kit (Invitrogen, CA) according to standard protocols. Gene expression was detected by real-time PCR analysis using SYBR DimerErase on the 7900HT system. U6 and GAPDH were utilized as normal controls for miRNA and mRNA and lncRNA, respectively. The fold change was calculated by the 2^−ΔΔCT^ method. The sequences of our primers were as follows: miR-3128, 5'-AAC GAGACGACGACAGAC-3' and 5'-TCTGGCAAGTAAAAAACTCTCAT-3'; LINC01410, 5'-GTGACAAGAATGGCCCAAGC-3' and 5'-ACTGTGCACCTGTTACAC CA-3’; U6, 5'-AACGAGACG ACGACAGAC-3' and 5'-GCAAATTCGTGAAGCGTTCCATA-3'; E-cadherin, 5’-TGCCC AGAAA ATGAA AAAGG-3’; and 5'-GTGTA TGTGG CAATG CGTTC-3’; N-cadherin, 5’-CCGGA GAACA GTCTC CAACTC-3’ and 5’-CCCAC AAAGA GCAGC AGTC-3’

### Cell growth, invasion and cell cycle assays

Cell growth was measured by the CCK-8 assay (Dojindo, Japan). Cells were plated in 96-well plates and were determined on days 0, 1, 2 and 3. Ten CCK-8 reagent was added to each well, and absorbance detection (OD) at 450 nm was detected on a microplate reader. Cells were cultured on the upper part of the Transwell extracellular matrix-coated chamber in serum-free medium. Culture medium containing 20% FBS was added to the lower chamber. After 2 days, the cells were stained with crystal violet and counted. For the cell cycle assay, cells were harvested and fixed with cold ethanol. Then, the cells were stained with propidium iodide and treated with RNase A and analyzed by flow cytometry.

### Luciferase reporter assay

To establish one luciferase reporter plasmid, the LINC01410 3’UTR fragment containing miR-3128 binding sites was cloned into the pMir-Report vector. A mutant construct was generated by substituting seed regions of the miR-3128 binding site and was named LINC01410 mut-3’UTR. Cells were cotransfected with the LINC01410 mut-3’UTR and LINC01410 wt-3’UTR luciferase reporter vectors and scramble or miR-3128 mimic by Lipofectamine 2000 (Invitrogen). Luciferase activities were calculated by the Dual-Luciferase Reporter System (Promega).

### Statistical analysis

The results are presented as the mean ± standard deviation. Student's t-test was utilized to assess the statistical significance of different groups via SPSS 17.0. Spearman’s rank correlation was used to determine correlation between LINC01410 and miR-3128. A P value < 0.05 was considered statistically significant.
